# The Pay of the Nurse. VII.—Causes of Low Salaries
*Previous articles appeared March 5, 12, 26, and April 2, 9, and 16.


**Published:** 1921-04-23

**Authors:** 


### THE MATRONS' AND SISTERS' DEPARTMENT.

### THE PAY OF THE NURSE.*

### VII.—Causes of Low Salaries.

At one end of the scale in the nursing profession we find women who have dedicated themselves to sacrifice for others, and who are indifferent to money either because they have learned to be content with little, or because they have already an independent income. At the other end are women whose standard of education and professional qualification is very low and who cannot command high remuneration because, to speak quite frankly, their services are not worth it. In both cases the effect is the same. A small salary is offered and accepted. The main body of nurses which stands between the two extremes suffers in consequence.

There can be no denying, we fear, that the higher service a man or woman renders to the community the less chance there is of his obtaining adequate reward in money. Some of the priceless research work now being carried on in the hospitals is done on far less than a day labourer's wage. The curate often earns less than the pig-keeper, and the author than the printer. The incentive to work of an enduring or ennobling character is so strong that it is tacitly allowed to be its own reward. In itself it buys what money cannot buy—an abiding interest in life and a sense of harmony in the performance of life's duties. Men and women will always be found freely dedicating themselves to forward the high aims of voluntary institutions for the sick.

Yet it is not in the end for the good of institutions that they should take advantage of the self-forgetfulness of their staff. Unsalaried or under-salaried posts have often proved very costly luxuries, and the circumstance that services can be obtained at a lower rate than is commercially sound is now seldom allowed to weigh with committees in making appointments. Rather it is a point of honour to pay what is generally considered just. Nothing has proved of greater service in helping managers to ascertain what they ought to pay their staff than the suggestions put forward as a minimum by the College of Nursing. The work of both voluntary and municipal institutions will increase in value to the community in proportion as they study to secure for all their employees a fair rate of remuneration and security for the future. Concerted and continued action is necessary to bring this about and to abolish the exploitation of the many on account of the self-sacrifice of the few.

The numerous incompetent and ill-educated women to be found in the ranks do great mischief to the nursing profession. They depress the economic value of nursing and make it difficult for a fair minimum rate of pay to be enforced. Every profession, at the outset of its organised existence, is hampered by members who are imperfectly instructed or are lacking in some one or other of the qualities requisite for efficiency in their duties. Under registration such as are glaringly unsuited to their work may be gradually eliminated. But the main hope lies in refusing entry to women who will not bring credit to the nursing profession. If ignorant to such an extent that they cannot grasp the elements of their craft, they should not be admitted to training, and the establishment of some simple form of entry examination is the only method by which they can be excluded. If careless and incompetent in the discharge of their practical duties they can more easily be weeded out by the central nursing authority than in the final examination at their training-schools, where rejection after three years' work is not very usual.

It is important to recognise the fact that the nursing service as a whole cannot be brought to the requisite level of satisfactory pay until it reaches a stage in which a stable level of efficiency has been reached by all its members. In this way it can plainly be seen that the ceaseless work of the College of Nursing in promoting sounder educational ideals is building the foundations at the same time of increased prosperity for nurses as a body. Not merely in opening up well-paid posts for women of higher education, but in studying to evolve a better educated, better trained, and therefore better paid rank and file, the College is building for the future.

* Previous articles appeared March 5, 12, 26, and April 2, 9, and 16.

